# Chemical Investigation of a Biologically Active *Schinus molle* L. Leaf Extract

**DOI:** 10.1155/2019/8391263

**Published:** 2019-07-28

**Authors:** Stefania Garzoli, Valentina Laghezza Masci, Elisa Ovidi, Giovanni Turchetti, Daniele Zago, Antonio Tiezzi

**Affiliations:** ^1^Department of Drug Chemistry and Technology, Sapienza University, Rome, Italy; ^2^Department for the Innovation in Biological, Agrofood and Forestal Systems, Tuscia University, Viterbo, Italy

## Abstract

The pepper tree *Schinus molle* L. is an evergreen ornamental plant belonging to the Anacardiaceae family, native to South America and widespread throughout the world. It has biological activities and is used in folk medicine. This paper aims to contribute to a deeper knowledge of its chemical composition and biological properties. *S. molle* leaf extracts were obtained by sequential extraction with solvents of different polarities and subsequently tested on the HL-60 human leukaemia cell line to define a possible cytotoxic activity. Among the investigated extracts, the petroleum ether extract revealed a high cytotoxic activity, and its chemical composition was further investigated. By a silica column chromatography, eight fractions were obtained, and their compositions were determined by GC-MS analysis. Compounds and relative abundance differed widely among the fractions; sesquiterpenes resulted the main component and alcoholic sesquiterpenes the most abundant.

## 1. Introduction

“Pink pepper” is the common name of *Schinus molle* L., a plant belonging to the Anacardiaceae family, which originates in South America and nowadays distributed throughout the world [1, 2]. *S. molle* is a dioecious plant with a peppery smell lanceolate leaves, pendulous branches with yellowish-white flowers arranged in clusters, and coral-red fruits in the size of peppercorns [3]. Some studies investigated different parts of the plant such as leaves, mature as well as immature fruits, stems, and branches to define the molecular composition and the related biological activities [4–8]. *S. molle* leaf essential oils showed antibacterial and antifungal activities [9–12], and anticancer and anti-inflammatory properties were also reported [13–16].

In view of the growing interest in the application of the natural products in the food and pharmaceuticals and in order to contribute for a deeper knowledge of biological properties of *S. molle*, we report here our findings concerning the molecules possibly involved in the cytotoxic activity.

## 2. Materials and Methods

### 2.1. Plant Collection and Extract Preparation

Fresh leaves of female *S. molle* (SML) plant were collected at “Angelo Rambelli” Botanical Garden (Tuscia University, Viterbo, Italy) in October 2017, out of the flowering period. The plant materials were washed with distilled water, air-dried in a shaded area for 2–4 days, and carefully hand-selected to separate the leaves, which were kept in a plastic container and frozen at −80°C for freeze-drying. After lyophilisation, the dried material was chopped and stored at 4°C until use. Dried leaves (3.5 g) were extracted using three solvents (100 mL each) of increasing polarity such as petroleum ether, diethyl ether, and acetone in a Soxhlet apparatus. After 6 extraction cycles for each solvent (SML1: petroleum ether extract; SML2: diethyl ether extract; SML3: acetone extract), the solid extracts were obtained by rotary evaporation (RV 08-VC, IKA, USA) at low pressure and 40°C of temperature.

The yield percentage from the dried extracts was calculated using the following equation:

(1) $$\begin{array}{c} \text{yield percentage}=\frac{\left(W1*100\right)}{W2}, \end{array}$$

where *W*1 is the dry weight of the extract after evaporating the solvent and *W*2 is the weight of the dried leaves.

### 2.2. Cell Line and Culture Conditions

The human promyelocytic leukaemia cell line (HL-60) was purchased from American Type Culture Collection (ATCC). This cell line was cultured in RPMI 1640 containing 1 mmol/L L-glutamine, supplemented with 10% (v/v) fetal bovine serum (FBS) and 1% (w/v) penicillin/streptomycin, and maintained at 37°C in humidified 5% CO_2_ incubator. Three times a week, cells were diluted to maintain a density of 5 × 10^5^/mL and harvested in the exponential phase of growth.

### 2.3. Cell Viability Assay

To analyse the cell viability, MTT assay was used. This is a colorimetric assay able to measure the reduction of 3-(4,5-dimethyl-2-thiazolyl)-2,5-diphenyl-2H-tetrazolium bromide (MTT) by mitochondrial succinate dehydrogenase. MTT enters the cells and passes into the mitochondria where it is reduced to an insoluble, coloured, formazan product. The formazan crystals inside the cells are then solubilised with an organic solvent (DMSO) and the released, solubilised reagent is measured spectrophotometrically [17]. HL-60 cells were seeded in flat-bottom 96-well plates at a density of 1 × 10^4^ cells/well and treated with ten 2-fold dilutions (250 *μ*g/ml–0.485 *μ*g/ml) for 24 h. As negative control, untreated cells were added. Vinblastine (V1377, Merck, Germany) was used as positive control with eleven 2-fold dilutions (10^−6^ M to 10^−9^ M) for 24 h. For solvent control, cells were treated with 0.1% DMSO for 24 h.

Following treatment incubation time, the media was carefully aspirated and 100 *μ*L of MTT solution (0.5 mg/mL) added to each well; after 3 h of incubation at 37°C, the formazan crystals were dissolved with 100 *μ*L of DMSO. Plates were read by a Sunrise Tecan microplate reader, and the absorbance was measured at 595 nm. The amount of colour produced is directly proportional to the number of viable cells.

Data showed in the present paper represented the average values of three independent measurements. EC_50_ value (effective concentration value) was defined as the concentration of treatment at which there was 50% growth inhibition of cell proliferation.

### 2.4. Low-Pressure Column Chromatography

SML1 (0.296 g) was fractionated by low-pressure column chromatography (LPLC) using silica gel 60H (0.04–0.063 mm) (Macherey-Nagel, Germany) as stationary phase [18]. The silica powder was added in a beaker to the mobile phase solvents used (hexane/diethyl ether, 80/20) and stirred to obtain a pourable slurry.

The stationary phase was packed into a glass column (*h* = 25 cm; *d* = 2.5 cm). A layer of sand was placed at the top of the silica gel, and the sample was dissolved in a small amount of the initial mobile phase and gently applied to the top of the column bed. Mixtures (v/v) of hexane (HX), diethyl ether (DE), ethyl acetate (EA), and ethanol (EtOH) in increased polarity mode were used for the elution (HX/DE 80/20; HX/DE 70/30; HX/DE 65/35; HX/DE 60/40; HX/DE 50/50; HX/DE 40/60; DE 100; DE/EA 90/10; DE/EA 80/20; DE/EA 50/50; EA 100; EA/EtOH 50/50), and the mobile phase was dropped under gravity. Fractions were collected manually, and the yield percentage of each fraction was calculated considering the dry weight of fraction after evaporating the solvent and weight of SML1.

### 2.5. Thin-Layer Chromatography

Fractions obtained by LPLC were analysed by thin-layer chromatography (TLC) with the purpose of defining the ones with similar chromatographic profiles and to group them. Precoated TLC sheets (20 × 20 cm Alugram® Xtra SIL G/UV254 sheet plates, Macherey-Nagel, Germany) were used for TLC. Samples were spotted on the bottom of the plate (approximately 1.5 cm above edge) by a Hamilton microsyringe. Afterwards, the TLC plates were placed in a presaturated chamber with solvents mixture HX/DE (80/20) and were let to develop. The plates were removed from the chamber before the solvents mixture reached their tops and allowed to dry under the hood and the separated spots were inspected by UV lights (254 nm and 365 nm, respectively). For terpenoid compounds detection, TLC plates were sprayed with vanillin/sulphuric acid (4.0%) and the fractions with a similar chromatographic profile were combined.

### 2.6. GC-MS Analysis

The GC-MS analyses (two repetitions for each sample) were performed using a PerkinElmer gas chromatograph, Model Clarus 500, equipped with a polar capillary column (Restek Stabilwax fused silica) directly coupled to a mass selective detector of the same company operated in the EI mode (70 eV).

Helium was used as the carrier gas at a flow rate of 1 mL min^−1^. The operating conditions were as follows: injector temperature 250°C and the oven temperature program: isothermal at 60°C for 5 min, ramped to 220°C at a rate of 5°C min^−1^, and finally isothermal at 220°C for 15 min. The dried extract and each fraction were dissolved in 1 ml of methanol and the injection volume for all samples was 2 *μ*L. The injector split ratio was 1 : 10. The extract constituents were identified by comparison of their linear retention indices (relative to C8–C30 aliphatic hydrocarbons, Ultrasci injected directly into GC injector under the same operating conditions reported above) with literature values and their mass spectra were compared with those from Wiley and NIST libraries. GC-FID analysis was performed under the same experimental conditions using the same column as described for the GC-MS measurements. The FID temperature was 280°C. Percentage area values were obtained electronically from the GC-FID response, without the use of an internal standard or correction factors.

### 2.7. Statistical Analysis

The EC_50_ values from three independent graphic plots of the dose-response curve were expressed as means ± standard error of the mean (SEM). Statistical analysis was performed by one-way analysis of variance (ANOVA test) using a Stat-Plus software (AnalystSoft VC 2009) with the threshold of significance set at *p* < 0.05.

## 3. Results and Discussion

### 3.1. *S. molle* Extract Yields and Its Antiproliferative Activity

The yields of the three extracts obtained from *S. molle* leaves are reported in Table 1. Petroleum ether extract (SML1) showed a yield about 8%, while the diethyl ether extract (SML2) and acetone extract (SML3) had a minor yield (1.4% and 2.0%, respectively). SML1, SML2, and SML3 were investigated by MTT assay to define the antiproliferative activities on human leukaemia cells (HL-60 cells). Each sample showed dose-dependent inhibition effects after 24 h of treatment. EC_50_ of the extracts exhibited values not exceeding 30.0 *μ*g/mL. The petroleum ether extract (SML1) showed a stronger antiproliferative activity with an EC_50_ value of 13.3 ± 6.1 *μ*g/mL (Table 1).

### 3.2. LPLC Fractions

According to the results obtained by MTT assay, SML1 was fractionated by LPLC to investigate its components, and after comparison by TLC, those having a similar chromatographic profile were reunited in eight final fractions (F1–F8) whose yields are reported in Table 2.

### 3.3. GC-MS Analyses

SML1 was analysed by GC and GC-MS analytical techniques, revealing that the extract consisted of 31 components (Table 3). The composition of the extract was dominated by sesquiterpene alcohol (56.41%), sesquiterpene hydrocarbons (25.17%), and a lower percentage of monoterpene hydrocarbons (4.18%). The major identified component from the sesquiterpenes family was elemol (46.28%) followed by germacrene D (18.10%); on the contrary, sabinene was detected as the major constituent of the terpenes group (1.65%). The presence of the squalene was relevant reaching 10.60%. Deveci et al. [19] reported germacrene D as a major constituent of *S. molle* leaf hexane extract. The composition of *Schinus* may differ by seasons, regions from which the plant material was collected, and by solvents and parts (leaves or fruits) of the plant used for the extraction [20].

The GC-MS analysis of the fractions obtained from the extract consisted of a mixture of different classes of identified compounds, which are reported as separate columns in Table 3. The main components of fraction F1 were *δ*-cadinene (50.58%), germacrene D (20.70%), and dehydroxy-isocalamendiol (18.45%). Fraction F2 was mainly characterised by elemene (72.16%) whereas dehydroxy-isocalamendiol (73.85%) was the most abundant in fraction F3. Fraction F4 was almost constituted by squalene (93.85%) while fraction F5 was made up of two components: *β*-thujene (49.88%) and *β*-terpinene (50.12%), present in similar percentage ratio. In fraction F6, *β*-thujene was the main component (67.97%) followed by sabinene (23.37%). Fraction F7 was almost entirely constituted by alloaromadendrene (95.21%) while the fraction F8 by *β*-eudesmol (65.87%). Other works reported the identification of high amounts of sesquiterpenes in the analysed extracts [21].

### 3.4. Antiproliferative Activity of LPLC Fractions

The fractions obtained by LPLC were tested by MTT and the results are reported in Table 2. Each of the eight fractions showed a relevant antiproliferative activity against human leukaemia cells with EC_50_ values ranging from 143.5 *μ*g/mL to 3.8 *μ*g/mL. Fraction F4, in which squalene represents 93.85%, was found to be the most active (EC_50_: 3.8 *μ*g/mL). Squalene is a triterpene molecule able to inhibit chemically induced colon, lung, and skin tumorigenesis in rodents and is investigated as an adjuvant cancer therapy [22–26].

Fractions F5 and F6, with a similar antiproliferative activity (EC_50_: 4.1 *μ*g/mL; 5.4 *μ*g/mL respectively), were mainly characterised by components belonging to the family of terpenes (*β*-thujene, *β*-terpinene, and sabinene). In particular, *β*-thujene from several plants and/or essential oils [27] has been described to be important for its antioxidant and antimicrobial activities.

*β*-Terpinene is one of three isomeric monoterpenes differing in the positions of their two double bonds (*α*- and *γ*-terpinene being the others). Terpinenes are three isomeric hydrocarbons that are classified as terpenes. Terpenes, one of the most extensive and varied structural group of compounds occurring in nature, display a wide range of biological and pharmacological activities and strong antioxidant property [28]. Wiart [29] has reported that the terpenes compromise the cell cycle in different types of malignancies by targeting the mitotic spindle and cyclins and thus triggering apoptosis.

Fractions F3 and F5 (EC_50_: 5.5 *μ*g/mL; 6.5 *μ*g/mL, respectively) are characterised by the presence of compounds belonging to the sesquiterpenes group. Dehydroxy-isocalamendiol is the main component of fraction F3 (73.85%) and is also present in fraction F1 (18.45%). This compound has been reported in essential oil from leaves and fruits of *S. molle* grown in Saudi Arabia [30].

In fraction F1, germacrene D was present; it is known that germacrene D has strong antimicrobial activity [31], and de Oliveira et al. [32] found some oils having anticancer effects in various cancer cell lines, with germacrene D as one of the main compounds.

## 4. Conclusions

In our study, the chemical composition of the petroleum ether extract (SML1) from leaves of *Schinus molle* L. and its eight fractions were determined by GC-MS and tested on HL-60 human leukaemia cells. The major component of the extract was elemol followed by germacrene D and squalene. The obtained fractions were abundantly rich in secondary metabolites such as monoterpenes and sesquiterpenes. The MTT results of the fractions demonstrated a relevant antiproliferative activity against human leukaemia cells. Considering both biological and chemical results, we hypothesise that the presence of compounds belonging to the family of terpenes (monoterpenes and sesquiterpenes) could be responsible for the activity against human leukaemia cells (HL-60). In the next future, the molecules here identified as possible responsible for anticancer activity could open perspectives to formulate new chemotherapeutic agents of natural origin.

## Figures and Tables

**Table 1:** Obtained yields and antiproliferative activities of *S. molle* leaf extracts (SML1, SML2, and SML3) against human leukaemia cells (HL-60) expressed by EC_50_ (mean values ± SEM).

Extracts	Yields (%)	EC_50_ (*μ*g/mL)
SML1	8.4	13.3 ± 6.1
SML2	1.4	23.8 ± 3.8
SML3	2.0	27.4 ± 7.0

**Table 2:** SML1 fractions obtained by LPLC and tested on human leukaemia cells (HL-60).

LPLC fractions	Yields (%)	EC_50_ (*μ*g/mL)
F1	23.7	6.5 ± 0.9
F2	30.3	12.8 ± 1.3
F3	3.6	5.5 ± 0.4
F4	1.9	3.8 ± 0.3
F5	1.7	5.4 ± 0.3
F6	4.7	4.1 ± 0.3
F7	11.4	143.5 ± 34.1
F8	4.6	37.9 ± 10.7

Cytotoxic activity was expressed by EC_50_ (mean values ± SEM). Vinblastine was used as positive control (EC_50_ = 12.2 ± 1.3 nM).

**Table 3:** Chemical composition (%) of the petroleum ether extract (SML1) and fractions from female *S. molle* leaves.

Component	LRI^1^	LRI^2^	SML1^*∗*^	F1	F2	F3	F4	F5	F6	F7	F8
Sabinene	1112	1107	1.63	—	1.45	6.35	2.65	—	23.37	—	—
*β*-Thujene	1122	1117	0.72	—	1.04	4.45	2.46	49.88	67.97	—	—
(+)-4-Carene	1153	1149	0.43	—	—	0.92	0.56	—	8.66	—	2.15
*α*-Phellandrene	1168	1160	0.34	—	—	—	—	—	—	—	—
*β*-Terpinene	1210	1206	0.68	—	—	—	—	50.12	—	4.79	7.63
*γ*-Terpinene	1251	1244	0.20	—	—	3.35	0.48	—	—	—	—
Terpinolene	1296	1290	0.18	—	—	5.97	—	—	—	—	—
*α*-Copaene	1491	1487	0.37	—	—	—	—	—	—	—	—
*α*-Gurjunene	1532	1527	0.75	—	—	—	—	—	—	—	—
*β*-Ylangene	1580	1574	0.36	—	—	—	—	—	—	—	—
*β*-Elemene	1590	1583	1.40	1.78	—	—	—	—	—	—	—
*β*-Caryophyllene	1624	1619	2.22	3.40	—	—	—	—	—	—	—
*γ*-Elemene	1649	1641	1.10	0.94	—	—	—	—	—	—	—
Alloaromadendrene	1655	1649	0.31	—	—	5.11	—	—	—	95.21	—
*α*-Humulene	1672	1667	0.85	1.46	—	—	—	—	—	—	—
*γ*-Muurolene	1682	1676	0.21	1.42	—	—	—	—	—	—	—
Germacrene D	1720	1717	18.10	20.70	—	—	—	—	—	—	—
*δ*-Cadinene	1761	1758	1.60	50.58	—	—	—	—	—	—	—
*γ*-Cadinene	1768	1760	0.12	1.27	—	—	—	—	—	—	—
Bicyclogermacrene	1773	1769	0.40	—	—	—	—	—	—	—	—
Palustrol	1942	1936	0.46	—	—	—	—	—	—	—	—
Germacrene D-4-ol	2062	2058	2.40	—	3.47	—	—	—	—	—	—
Elemol	2079	2076	46.28	—	72.16	—	—	—	—	—	—
Viridiflorol	2093	2091	1.18	—	6.01	—	—	—	—	—	—
Dehydroxy-isocalamendiol	2138	—	1.41	18.45	—	73.85	—	—	—	—	—
Spathulenol	2140	2136	0.35	—	0.45	—	—	—	—	—	—
*γ*-Eudesmol	2184	2176	0.68	—	2.70	—	—	—	—	—	24.35
*α*-Eudesmol	2240	2232	1.69	—	6.06	—	—	—	—	—	—
*β*-Eudesmol	2255	2249	1.96	—	6.66	—	—	—	—	—	65.87
Squalene	2853	2865^+^	10.60	—	—	—	93.85	—	—	—	—
Total			98.98	100.00	100.00	100.00	100.00	100.00	100.00	100.00	100.00
Monoterpene hydrocarbons			4.18	—	2.49	21.04	6.16	100.00	100.00	4.79	9.78
Sesquiterpene hydrocarbons			25.17	78.15	—	5.11	—	—	—	95.21	90.22
Sesquiterpene alcohol			56.41	18.45	97.51	79.85	—	—	—	—	—
Bicyclic sesquiterpenes			2.62	3.40	—	—	—	—	—	—	—
Triterpenes			10.60	—	—	—	93.85	—	—	—	—

^1^Linear retention indices measured on polar column; ^2^linear retention indices from the literature; ^+^normal alkane RI; ^*∗*^female petroleum ether extract; F1 to F8: fractions from the petroleum extract; —, not available.

## Data Availability

The data used to support the findings of this study are included within the article.
